# Operationalizing perioperative clearance reserve: a reserve-load interaction model for neurocognitive outcomes in older surgical patients

**DOI:** 10.3389/fmed.2026.1921232

**Published:** 2026-09-16

**Authors:** Budanbaila La, Yanyan Bai, Yi Qiu, Ling Wang

**Affiliations:** 1 Department of Anesthesiology, The Affiliated Hospital of Inner Mongolia Medical University, Hohhot, Inner Mongolia, China; 2 Department of Anesthesiology, Hohhot Maternal and Child Health Care Hospital, Hohhot, Inner Mongolia, China; 3 Department of Anesthesiology, The Second Affiliated Hospital of Inner Mongolia Medical University, Hohhot, Inner Mongolia, China

**Keywords:** anesthesiology, glymphatic system, mechanotransduction, meningeal lymphatics, perioperative brain health, perioperative medicine, perioperative neurocognitive disorders, postoperative delirium

## Abstract

Older patients who undergo similar operations and receive comparable anesthetic care do not always recover cognitively in the same way. Inflammation explains an important part of perioperative brain injury, yet it does not fully account for why the response settles quickly in one patient and persists in another. We suggest that this difference may reflect an interaction between the patient's preoperative capacity for brain fluid and solute clearance and the load imposed by surgery and anesthesia. In the proposed model, baseline clearance reserve is a continuous latent phenotype informed primarily in initial human studies by structural and transport measures of the glymphatic and meningeal lymphatic systems; molecular-cellular features are evaluated in nested or translational studies. Postoperative change is treated separately and followed as a recovery trajectory within each patient. Perioperative load is measured across mechanical-fluid, inflammatory, and surgical domains. The main statistical test is the interaction between baseline reserve and a prespecified load measure after adjustment for baseline confounders and for perioperative predictors not used to construct that load measure. Mechanotransduction may connect physical load with altered clearance. PIEZO1 is included as a tractable candidate within this pathway, rather than as a required cause. Initial human validation should focus on adults aged 65 years or older undergoing elective major noncardiac, nonintracranial surgery; cardiac and intracranial procedures require separate analyses. The theory can be tested by asking whether preoperative clearance measures add information beyond established risk factors, whether reserve modifies the effect of perioperative load, and whether postoperative clearance trajectories are related to neuroimmune and cognitive outcomes. This approach makes clearance reserve measurable while keeping it distinct from a clinical score.

## 1 Introduction

Perioperative neurocognitive disorders include postoperative delirium, delayed neurocognitive recovery, and postoperative mild or major neurocognitive disorder. In older surgical patients, even a short period of cognitive disturbance may delay mobilization, interfere with rehabilitation, prolong hospital stay, or bring an unrecognized vulnerability to clinical attention (1–3). Prediction remains difficult. Patients of a similar age can undergo comparable operations under similar anesthetic conditions and still follow quite different cognitive courses.

Surgery can trigger systemic inflammation, disturb the blood-brain barrier, activate microglia, injure neurons, and impair synaptic plasticity (4–8). These processes explain much of the acute response. They are less successful at explaining recovery. The same inflammatory burden may settle within days in one patient but remain biologically active in another. This suggests that vulnerability depends not only on the size of the insult, but also on the brain's capacity to restore fluid, immune, and metabolic balance.

Glymphatic transport and meningeal lymphatic drainage are plausible parts of that capacity. Together they support cerebrospinal fluid-interstitial fluid exchange, solute removal, immune surveillance, and drainage toward deep cervical lymph nodes (9–15). Several components deteriorate with age (16–19). Meningeal lymphatic function also shapes microglial responses in animal models (20). In animal studies, impaired brain lymphatic drainage increases susceptibility to surgery-related neuroinflammation and cognitive dysfunction, whereas better drainage improves postoperative findings in aged animals (21, 22).

Wang et al. recently described the glymphatic-meningeal lymphatic system as a central clearance reserve and proposed that perioperative inflammatory load may exceed available capacity (23). We take that biological idea a step toward testing. Clearance reserve is defined before surgery, postoperative failure is measured as change over time, perioperative load is recorded directly, and the main hypothesis is expressed as a statistical interaction. The same structure also allows the theory, and its mechanotransduction branch, to be rejected if the predicted findings do not appear.

## 2 Scope, population, and temporal definitions

The primary human population is adults aged 65 years or older who undergo elective major noncardiac, nonintracranial surgery. This age boundary follows perioperative brain-health guidance and the 2025 American Society of Anesthesiologists practice advisory for older adults (2, 3). It is a practical enrollment criterion, not a biological dividing line. Age should still be analyzed as a continuous variable, with sensitivity analyses for nonlinear effects.

Cardiac surgery involving cardiopulmonary bypass and intracranial surgery should be kept out of the initial derivation cohort. Cardiopulmonary bypass adds embolic exposure, hemodilution, nonpulsatile flow, and a marked inflammatory response. Intracranial procedures may directly alter brain tissue, cerebrospinal fluid pathways, and intracranial pressure. Both groups warrant study, but each needs a load definition suited to its own physiology.

Measurements are organized across clinically recognizable periods. Baseline assessment takes place 7–30 days before surgery. The intraoperative period runs from anesthetic induction to discharge from the operating room. The first 72 postoperative hours capture the acute response, while delirium surveillance continues through postoperative day 7 or hospital discharge. Delayed neurocognitive recovery is assessed up to and including day 30. Postoperative mild or major neurocognitive disorder is evaluated after day 30 through 12 months, with planned assessments at 3 and 12 months. Under current nomenclature, cognitive decline occurring within 30 days is described as delayed neurocognitive recovery, whereas mild or major neurocognitive disorder diagnosed after day 30 through 12 months is termed postoperative neurocognitive disorder (1). Delirium remains a separate outcome because it fluctuates, requires different assessment methods, and usually occurs earlier.

Clearance reserve refers to capacity present before exposure: the ability of the neurovascular, glymphatic, and meningeal lymphatic systems to tolerate and recover from perioperative load. Postoperative clearance dysfunction describes what happens after that exposure. The outcome cannot be used to define either variable. Otherwise, low reserve would be inferred from PND and then used to explain the same PND, creating a circular argument.

## 3 Structured evidence mapping and appraisal

We used a structured narrative evidence map to assemble the argument. PubMed and the reference lists of key publications were searched through 23 August 2026. Search terms covered perioperative neurocognitive disorder, postoperative delirium, glymphatic and meningeal lymphatic function, aging, clearance, mechanotransduction, PIEZO1, diffusion tensor imaging along the perivascular space (DTI-ALPS), cerebrospinal fluid flow, surgery, anesthesia, and dementia. Papers were grouped according to the causal link they addressed and whether the evidence came from perioperative human studies, perioperative animal work, nonperioperative human research, or mechanistic animal and cellular experiments.

This classification draws on the Oxford Centre for Evidence-Based Medicine principle of evidence directness (24). It is not a formal GRADE assessment. The literature addresses different mechanistic questions and includes varied species, operations, tracers, imaging surrogates, sampling times, and cognitive tests. A pooled effect estimate would therefore be difficult to interpret. Table 1 instead shows the most relevant evidence for each link, how directly it applies to the perioperative question, and where uncertainty remains. Meta-analysis would become useful once several studies examine the same link with comparable exposure and outcome definitions.

**Table 1:** Directness and limitations of evidence for the proposed causal links.

Proposed link	Representative evidence	Directness	Interpretation	Main limitation
Aging and impaired clearance	Age-related decline in paravascular transport and meningeal lymphatic function (16, 17, 19)	Convergent animal and nonperioperative human evidence	Provides a plausible biological substrate	Human functional measures are indirect
Baseline drainage impairment and postoperative outcome	Deep cervical lymph-node ligation worsened surgery-related glymphatic dysfunction, inflammation, neuronal injury, and behavior (21)	Direct perioperative animal evidence	Supports greater susceptibility when drainage is impaired before surgery	Experimental ligation does not reproduce natural human aging
Improved drainage and postoperative outcome	Enhanced meningeal lymphatic drainage improved anesthesia- and surgery-related cognitive dysfunction in aged mice (22)	Direct perioperative animal evidence	Suggests that the pathway can be modified	Replication, cell specificity, and human translation are pending
Perioperative physiology and cerebrospinal fluid dynamics	Respiration, vascular pulsation, posture, and pressure influence cerebrospinal fluid and venous flow (25–28)	Mainly nonoperative human physiology	Makes mechanical-fluid load biologically plausible	Mediation of PND has not been demonstrated
PIEZO1 and meningeal lymphatic drainage	PIEZO1 regulates meningeal drainage; activation increased cerebrospinal fluid outflow and improved drainage in aged mice (29, 30)	Mechanistic animal evidence	Supports the choice of PIEZO1 as a candidate	A perioperative PND mechanism has not been tested
Human clearance measures and perioperative cognition	Indirect MRI surrogates are feasible, but no integrated longitudinal cohort exists (31, 32).	Indirect or nonperioperative human evidence; no integrated perioperative cohort	Association remains uncertain	Validity, confounding, and temporal resolution
Repeated surgery and accelerated aging or dementia	Observational results are mixed; a large matched cohort found no increase after common elective noncardiac surgery (33)	Human observational evidence	A causal relationship is not established	Indication bias, selection, baseline cognition, and time-varying confounding

Directness categories describe proximity to the human perioperative question and do not constitute a formal certainty-of-evidence grade. mLV, meningeal lymphatic vessel; CSF, cerebrospinal fluid; PND, perioperative neurocognitive disorders.

Evidence is strongest for age-related deterioration of clearance pathways and for worse postoperative findings in animals with pre-existing drainage impairment. Direct human perioperative evidence is much thinner. We found no older surgical cohort in which preoperative multimodal clearance, intraoperative mechanical and inflammatory exposure, serial postoperative clearance, and standardized cognitive outcomes were all measured together. Evidence for perioperative mechanotransduction is more limited again. The theory is presented with this uneven evidence base in view.

## 4 The reserve-load interaction model

The model asks whether perioperative load has a larger and more persistent cognitive effect in a patient with less baseline clearance capacity. Under this hypothesis, patients with greater reserve can tolerate more load before sustained clearance dysfunction and cognitive injury become likely. Patients with less reserve reach that region at a lower load (Figure 1).

**Figure 1:**
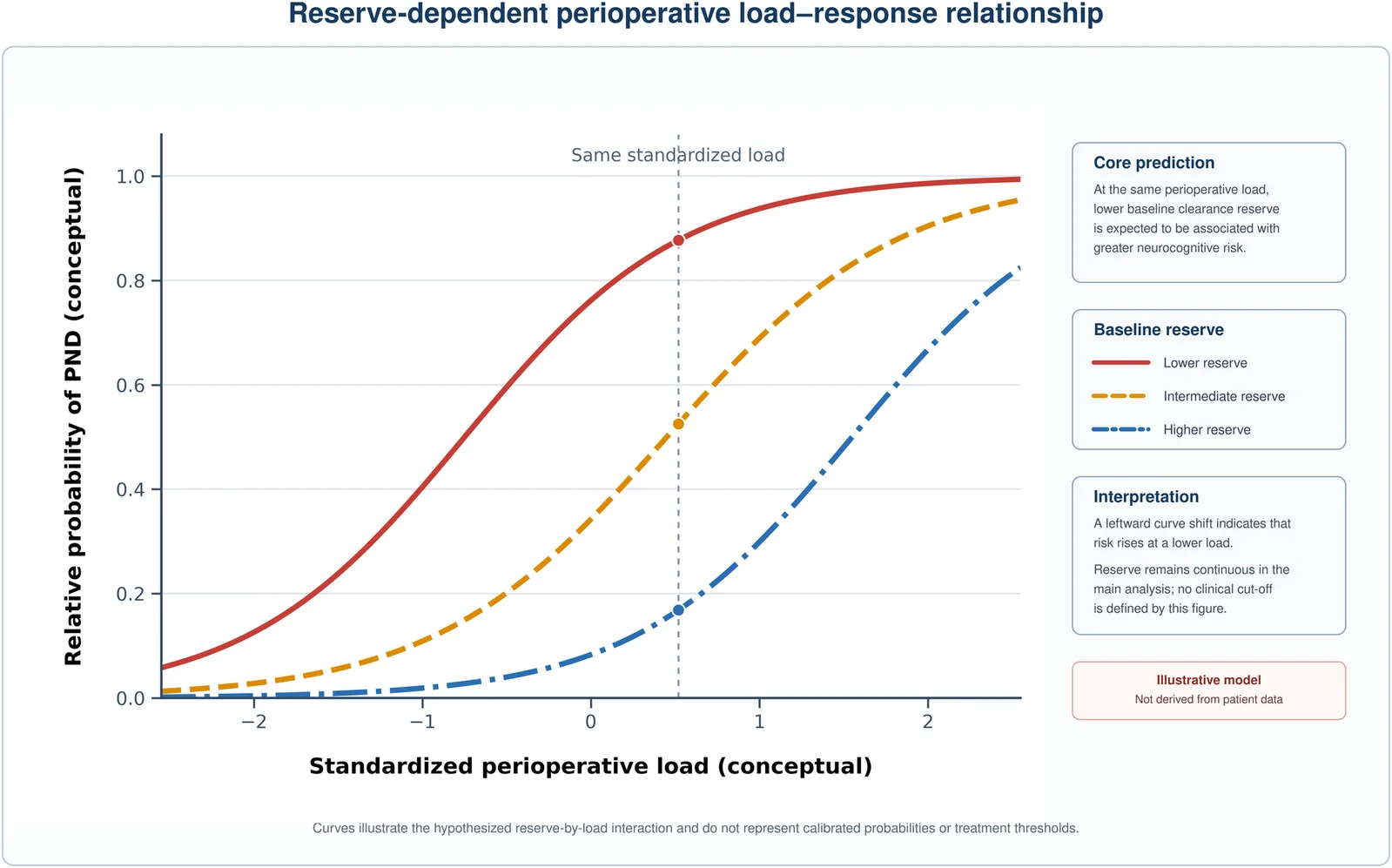
Reserve-dependent relation between standardized perioperative load and the conceptual probability of perioperative neurocognitive disorders at lower, intermediate, and higher levels of baseline clearance reserve. At the same standardized load, lower reserve is associated with greater conceptual risk. The nonparallel curves illustrate the hypothesized reserve-by-load interaction: the adverse load slope is steeper when reserve is lower and attenuated when reserve is higher. Curves are illustrative and are not derived from patient data, calibrated clinical thresholds, or treatment rules.

For participant i, let Rᵢ represent standardized baseline clearance reserve, Lᵢ a prespecified domain-specific load measure or composite, and Yᵢ a neurocognitive outcome assessed at a defined time. For a binary delirium outcome, the primary model is:

$$logit[P(Y_{i}=\;1)]\;=\;\alpha \;+\beta_{R}R_{i}+\beta_{L}L_{i}+\beta_{RL}(R_{i}\times L_{i})\;+\;\gamma {\;}^{\prime }C_{i}$$

The covariate vector Cᵢ contains baseline confounders that are not used to construct Rᵢ or Lᵢ: age, sex, education, baseline cognition, frailty, vascular disease, sleep disturbance, depression, and sensory impairment. Surgical severity and anesthetic, analgesic, or sedative exposure enter Lᵢ when they define the selected load; if they are not components of Lᵢ, they may be prespecified covariates. No variable used as an indicator of Rᵢ or as a component of Lᵢ is entered again unchanged in Cᵢ. With higher Rᵢ denoting better reserve, higher Lᵢ denoting greater load, and Yᵢ = 1 denoting delirium, the expected directions are *β*R < 0, *β*L > 0, and *β*RL < 0. The interaction coefficient tests whether the adverse effect of load becomes smaller as reserve increases.

Inflammation enters the analysis in more than one place. Preoperative inflammatory activity may indicate priming and can act as a covariate or modifier. The acute postoperative inflammatory response is time varying and may lie between surgery, clearance dysfunction, and cognitive change. Baseline reserve therefore serves as a moderator, while postoperative clearance change is evaluated as a possible mediator. Acute inflammation can be modeled as both a measured exposure and a mediator, depending on the timing of the samples (Figure 2).

**Figure 2:**
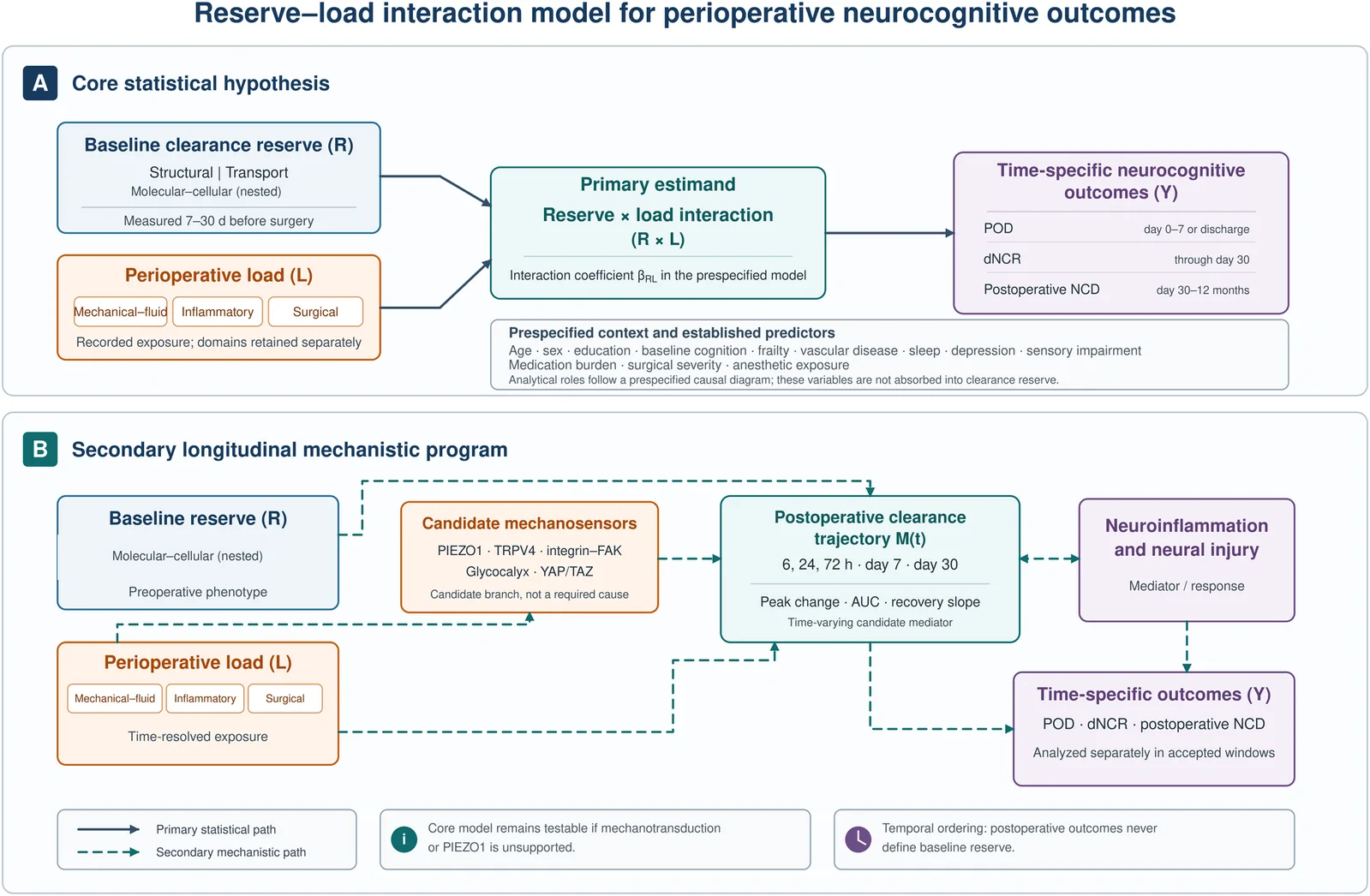
Statistical and mechanistic architecture of the reserve-load model. Panel **(A)** shows the core statistical hypothesis: baseline clearance reserve and measured perioperative load converge on the prespecified reserve-by-load interaction, which is tested against time-specific neurocognitive outcomes after accounting for established predictors and biopsychosocial context. Panel **(B)** shows the secondary longitudinal mechanistic program. Baseline reserve and perioperative load are related to postoperative clearance trajectories; the mechanical-fluid component may act through candidate mechanosensors, including PIEZO1, TRPV4, integrin-FAK signaling, the endothelial glycocalyx, and YAP/TAZ. Postoperative clearance dysfunction and neuroinflammation or neural injury are evaluated as time-varying mediators or responses. Solid arrows denote the primary statistical path; dashed arrows denote secondary mechanistic hypotheses. PIEZO1 is a candidate rather than a required cause. TRPV4, transient receptor potential vanilloid 4; FAK, focal adhesion kinase; YAP, yes-associated protein; TAZ, transcriptional coactivator with PDZ-binding motif.

The reserve-load model sits alongside inflammatory, vascular, and cognitive-reserve accounts (Table 2). Its added contribution should be judged quantitatively. If a baseline clearance phenotype fails to improve model fit, calibration, or performance in new patients, and if the interaction with load cannot be reproduced, the central hypothesis would need to be revised or abandoned. The operational definitions and prespecified falsification rules are summarized in Box 1.

**Table 2:** Relationship between established PND frameworks and the proposed reserve-load model.

Framework	Main explanatory factor	What it captures	What remains unclear	Place in the reserve-load model
Inflammation-centered model	Intensity and duration of systemic and central immune activation	Cytokine signaling, blood-brain barrier disturbance, and microglial activation after surgery	Why similar inflammatory exposure leads to different cognitive recovery	Inflammation may act as preoperative priming, perioperative load, and a time-varying mediator (4, 6).
Cognitive reserve	Network capacity to tolerate pathology	The modifying effects of education, prior function, and neural efficiency	Fluid, immune, and solute recovery are not measured directly	Treated as a parallel reserve and covariate rather than part of clearance reserve (34).
Vascular and blood-brain barrier model	Integrity of endothelium and the blood-brain interface	Entry of inflammatory mediators, vascular injury, and delirium biology	Removal and recovery after exposure are less well described	Vascular integrity contributes to the structural and molecular-cellular domains (8, 35).
Glymphatic-meningeal lymphatic imbalance	Balance between inflammatory burden and central clearance capacity	Persistence of perioperative inflammation when drainage is impaired	Human measurement, timing, and statistical testing remain limited	The closest biological precursor to the present model (23).
Reserve-load interaction model	Continuous baseline clearance reserve and recorded perioperative load	Variation in individual vulnerability and recovery	Prospective validation is needed; direct brain injury may follow another pathway	Defines variables, time points, an interaction coefficient, and criteria for rejection.

BBB, blood-brain barrier; PND, perioperative neurocognitive disorders.

Box 1 Operational definitions and falsification rules.

**Table:** 

Baseline construct	Clearance reserve is a continuous preoperative phenotype informed by structural, transport, and molecular-cellular features. It is measured independently of PND.
Postoperative response	Change in clearance and the subsequent recovery slope are time-varying responses and possible mediators.
Main hypothesis	The cognitive effect of a measured perioperative load changes across the distribution of baseline clearance reserve.
Study population	Adults aged 65 years or older undergoing elective major noncardiac, nonintracranial surgery.
Measurement windows	Baseline 7–30 days before surgery; intraoperative exposure; first 72 postoperative hours; delirium through day 7 or discharge; dNCR assessed by day 30; postoperative NCD assessed after day 30 through 12 months.
PIEZO1	A prioritized mechanotransduction hypothesis within the model, rather than a required cause.
When the model would fail	The model should be revised or rejected if reserve is not reproducible, adds no information beyond established predictors, or shows no consistent interaction with load.
Clinical limit	No current measure or threshold is ready to allocate treatment or prophylaxis.

dNCR, delayed neurocognitive recovery; NCD, neurocognitive disorder; PND, perioperative neurocognitive disorders.

## 5 Biological components of baseline clearance reserve

No single clinical measurement represents the full clearance system. Baseline reserve is therefore treated as a latent physiological phenotype informed by structural, transport, and molecular-cellular domains. The domains overlap biologically, but they should not be treated as interchangeable measurements. For initial human validation, the core phenotype should be built from observable structural and transport indicators. Molecular-cellular measures should be evaluated in nested or translational studies until technically validated for routine perioperative use.

Structural reserve describes the anatomical substrate that supports transport. Candidate measures include MRI-visible perivascular-space burden, white-matter disease, vascular stiffness, and, in specialized studies, meningeal lymphatic morphology or enhancement. Enlarged perivascular spaces do not measure flow directly and may reflect several vascular and interstitial processes (36, 37). Human meningeal lymphatics can be visualized with contrast-enhanced imaging, although repeated perioperative use and the link with functional drainage still require validation (38, 39).

Transport reserve concerns movement and exchange of fluid, a field in which measurement and mechanism remain debated (40). DTI-ALPS is noninvasive and can be added to a clinical MRI protocol, but it remains an indirect surrogate influenced by age, white-matter integrity, and vascular disease. In a large population sample, DTI-ALPS was more closely related to vascular measures than to amyloid deposition or perivascular-space burden (32, 41). It should not be read as a clearance rate. Phase-contrast MRI can measure cerebrospinal fluid velocity and pulsatility, yet pulsatility does not equal net solute removal. Respiration, arterial pulsation, venous pressure, and posture all change cerebrospinal fluid dynamics (25–28).

The molecular-cellular domain includes aquaporin-4 organization, lymphatic endothelial competence, neurovascular coupling, endothelial glycocalyx integrity, and immune homeostasis. Most of these features can currently be studied only in tissue or animal models. Plasma glial fibrillary acidic protein, neurofilament light, S100B, and cytokines indicate injury or biological response; they are not validated measures of clearance reserve. Their perioperative value is illustrated by the association between plasma neurofilament light and postoperative delirium (42); baseline cerebrospinal fluid Alzheimer disease biomarkers have also predicted postoperative cognitive dysfunction (43), although neither finding is a direct measure of clearance. Serial cerebrospinal fluid and plasma profiles may prove useful, although production, compartment exchange, renal elimination, and blood-brain barrier permeability all affect concentration. For now, this domain belongs in the biological model but not in a routine human score.

Postoperative clearance recovery is measured separately. It may appear as an early fall followed by recovery in an imaging surrogate, cerebrospinal fluid pulsatility, or a linked biomarker pattern. Peak change, area under the curve, and recovery slope can be estimated from measurements made at 6, 24, and 72 h, with later observations on day 7 and day 30 when feasible. These trajectories may mediate the effect of reserve and load on cognition, but they cannot be used retrospectively to define preoperative reserve.

## 6 Perioperative load: mechanical-fluid, inflammatory, and surgical domains

Perioperative load should be built from recorded exposures. The mechanical-fluid domain includes airway driving and plateau pressures, positive end-expiratory pressure, ventilation time, deviations in PaCO2, central venous pressure, venous congestion, fluid balance, body position, and the magnitude and duration of blood-pressure change from the patient's baseline. Each may influence vascular tone, respiratory-venous coupling, intracranial pressure, or cerebrospinal fluid movement. These perioperative determinants have been reviewed, but human evidence has not yet shown that they cause PND by reducing clearance (26–28, 31).

Inflammatory load reflects both the size and duration of immune activation. Tissue injury releases damage-associated molecular patterns and cytokines, changes endothelial and blood-brain barrier function, and can prime microglia (4, 6, 8, 44). A single postoperative concentration loses much of this information. Baseline C-reactive protein or cytokine levels describe preoperative priming, whereas postoperative rise and recovery describe the acute response.

Surgical load includes duration and anatomic site of the operation, tissue trauma, blood loss, transfusion, hypotension, hypoxemia, infection, and unplanned intensive care. These exposures should be examined by domain before they are combined. Two patients can receive the same composite score through clinically different patterns, and those patterns may affect clearance in different ways.

Load definitions must change with the operation. Embolic burden, bypass duration, nonpulsatile flow, hemodilution, and temperature shifts are central in cardiac surgery. During intracranial surgery, direct tissue manipulation and altered cerebrospinal fluid pathways may dominate. Separate analyses are needed for biological reasons, not simply to make the statistics cleaner.

## 7 Mechanotransduction as a candidate link

Mechanotransduction is the cellular conversion of physical force into a biochemical signal (45). It offers one route from mechanical-fluid exposure to altered clearance, although it is unlikely to explain every PND phenotype. Lymphatic and vascular endothelial cells, perivascular cells, astrocytes, microglia, and neurons respond to stretch, shear, matrix stiffness, and membrane tension (46–49). Aging changes tissue stiffness, vascular pulsatility, inflammatory tone, and response thresholds. The same physical exposure may therefore produce a different cellular response in an older system.

PIEZO1 was selected for practical and biological reasons. It is a mechanically activated, nonselective cation channel, and its curved membrane-dome architecture helps explain force sensing (50, 51). PIEZO1 senses shear and membrane tension, is present in vascular and lymphatic endothelial compartments, participates in flow-sensitive lymphatic development and remodeling, and can be manipulated experimentally. Animal studies show that PIEZO1 supports meningeal lymphatic drainage and that activation can increase cerebrospinal fluid outflow (29, 52). Agonism has also improved meningeal lymphatic coverage, drainage, and brain-cerebrospinal fluid perfusion in aged mice (30). These findings make PIEZO1 a reasonable candidate. They do not establish a perioperative PIEZO1-PND pathway.

PIEZO1 can influence calcium-dependent signaling, calcineurin-nuclear factor of activated T cells activity, endothelial adaptation, cytoskeletal remodeling, and immune-cell behavior (53–57). It remains unknown whether routine ventilation, positioning, and fluid shifts activate this pathway in the aging human meningeal lymphatic system strongly enough to affect cognition.

Other force-sensitive systems deserve equal attention, including transient receptor potential vanilloid 4, integrins and focal adhesions, cytoskeletal-nuclear coupling, endothelial glycocalyx signaling, and the yes-associated protein/transcriptional coactivator with PDZ-binding motif pathway (58–60). If PIEZO1 is uninvolved but another sensor carries the predicted load-clearance signal, the mechanotransduction branch would remain viable. If no mechanical pathway is found, inflammatory or vascular load could still interact with independently measured clearance reserve.

## 8 Operationalization in human perioperative studies

A practical starting design is a prospective multicenter cohort of patients aged 65 years or older undergoing elective major noncardiac, nonintracranial surgery (Table 3). Assessment 7–30 days before surgery would include a standardized cognitive battery, history of delirium or dementia, frailty, depression, sleep and obstructive sleep apnea risk, sensory impairment, education, vascular disease, medication burden, and noncontrast MRI. A core MRI protocol could include T1, T2/FLAIR, susceptibility, and diffusion imaging. DTI-ALPS and phase-contrast cerebrospinal fluid measurements can be studied in nested groups. Intrathecal or contrast-enhanced tracer studies are unnecessary for routine enrollment and should be reserved for separately approved research or clinical indications.

**Table 3:** Candidate operational measures, timing, analytical role, and limitations.

Domain or variable	Candidate measure	When measured	Role in the model	Caution in interpretation
Structural reserve	Perivascular-space burden; white-matter hyperintensity; vascular stiffness; research imaging of meningeal lymphatic vessels	7–30 days before surgery	Indicators of baseline latent reserve	Anatomy does not measure flow; contrast imaging is not routine
Transport reserve	DTI-ALPS; phase-contrast cerebrospinal fluid velocity or pulsatility	7–30 days before surgery; optional postoperative repeat	Indicators of baseline reserve and postoperative change	DTI-ALPS is indirect and sensitive to vascular factors; pulsatility is not net clearance
Molecular-cellular reserve	Aquaporin-4 organization, lymphatic endothelial function, neurovascular and glycocalyx measures	Mainly preclinical; selected research samples	Biological domain	No routine human measure has been validated
Mechanical-fluid load	Driving and plateau pressure, PEEP, PaCO2 and mean arterial pressure deviations, central venous pressure or congestion, position, and fluid balance	Continuously during surgery and early recovery	Exposure and interaction term	Keep the component data before deriving a composite
Inflammatory load	Baseline C-reactive protein; serial interleukin-6 and related markers; postoperative rise and recovery slope	Baseline and 6, 24, and 72 h after surgery	Priming covariate, exposure, and possible mediator	Concentration reflects production, distribution, and elimination
Neural injury response	Glial fibrillary acidic protein, neurofilament light, S100B, and electroencephalographic features	Baseline and during the first 72 h	Secondary mediator or outcome	These are not direct clearance measures
Dynamic clearance response	Within-patient imaging or physiological change, peak change, area under the curve, and recovery slope	First 72 h, day 7, and day 30	Possible mediator between reserve-load interaction and cognition	Postoperative change cannot define preoperative reserve
Neurocognitive outcomes	Confusion Assessment Method or CAM-ICU; standardized cognitive battery	Daily through day 7; day 30; 3 and 12 months	Separate clinical outcomes	Delirium, delayed recovery, and postoperative neurocognitive disorder should not be pooled indiscriminately
Biopsychosocial context	Education, frailty, sleep, depression, pain, sensory loss, social support, and medication burden	Baseline and follow-up	Confounders, modifiers, or parallel reserves	These variables should not all be absorbed into clearance reserve

CAM, Confusion Assessment Method; CAM-ICU, Confusion Assessment Method for the Intensive Care Unit; CRP, C-reactive protein; CSF, cerebrospinal fluid; CVP, central venous pressure; dNCR, delayed neurocognitive recovery; DTI-ALPS, diffusion tensor imaging along the perivascular space; EEG, electroencephalography; GFAP, glial fibrillary acidic protein; MAP, mean arterial pressure; mLV, meningeal lymphatic vessel; NCD, neurocognitive disorder; NfL, neurofilament light; PEEP, positive end-expiratory pressure.

Intraoperative physiology should be retained at high temporal resolution. Useful summaries include time-weighted driving pressure, PaCO2 deviation, hypotension relative to baseline, central venous pressure or venous congestion, fluid balance, anesthetic depth, anesthetic and opioid dose, blood loss, transfusion, and operating time. Delirium is assessed daily through day 7 or discharge. Selected physiological and molecular measures are repeated at 6, 24, and 72 h. Cognitive testing near day 30 provides the early recovery outcome, with further follow-up at 3 and 12 months where feasible (Figure 3).

**Figure 3:**
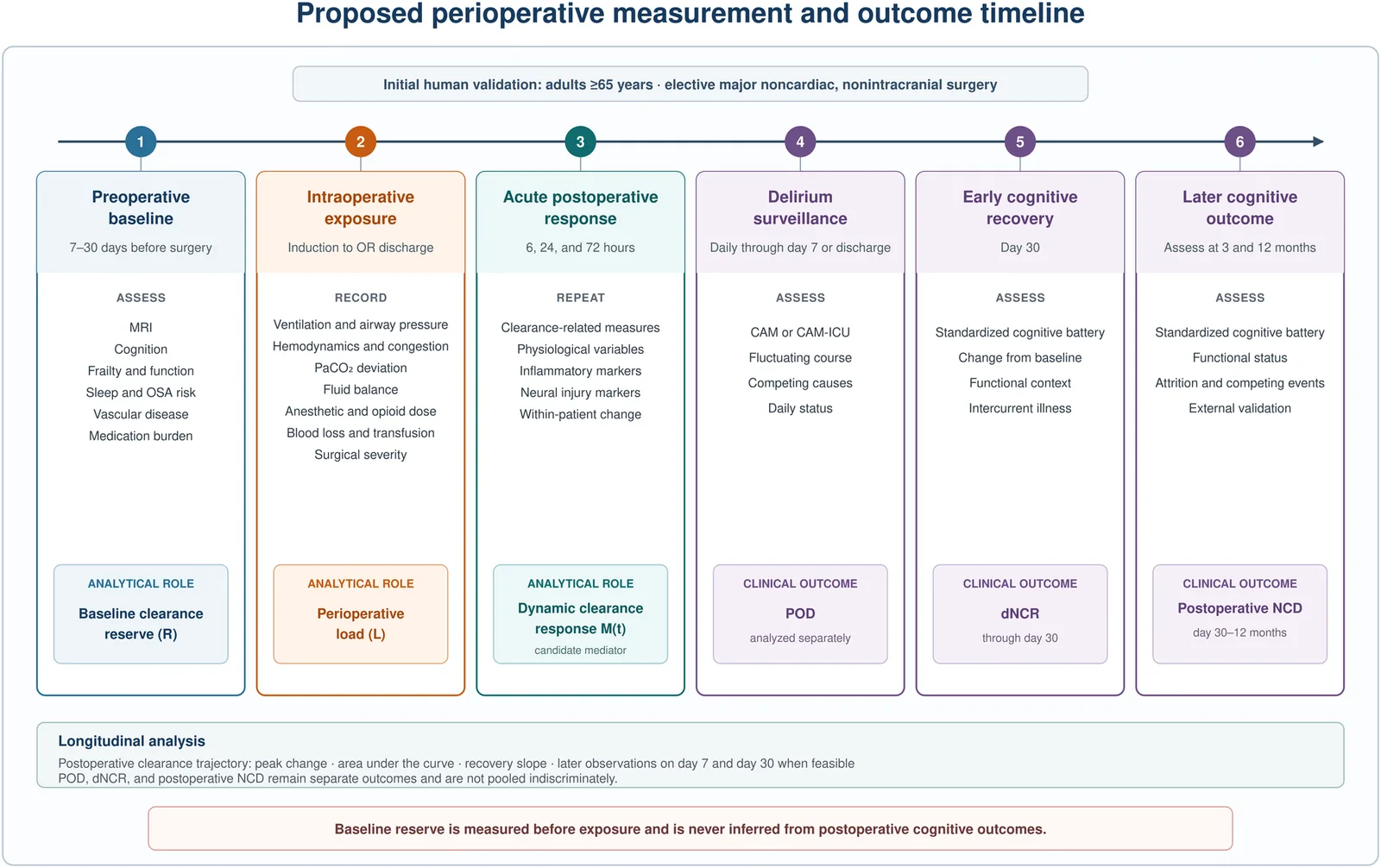
Proposed schedule of perioperative measurements and neurocognitive outcomes for initial human validation in adults aged 65 years or older undergoing elective major noncardiac, nonintracranial surgery. Baseline clearance reserve is assessed 7-30 days before surgery. Perioperative load is recorded from anesthetic induction to operating-room discharge. Clearance-related physiology, inflammation, and neural-injury markers are repeated at 6, 24, and 72 h, with later clearance observations on day 7 and day 30 when feasible. Postoperative delirium is assessed daily through day 7 or discharge, delayed neurocognitive recovery is assessed by day 30, and postoperative neurocognitive disorder is assessed after day 30 at 3 and 12 months. Baseline reserve is measured before exposure and is never inferred from postoperative cognitive outcomes.

Preoperative indicators can be combined as a continuous latent factor. Confirmatory factor analysis or a prespecified dimension-reduction method would estimate weights in a derivation cohort; those weights should be fixed before external validation. When the sample is too small for latent modeling, an equal-weight standardized composite offers a transparent exploratory option. It should be described as a research composite, not as a clinical index.

Reserve should remain continuous in the main analysis. Dividing patients into high and low groups would discard information and create arbitrary boundaries. Quantiles may help display the interaction or support a prespecified exploratory analysis. A treatment threshold would require independent calibration, assessment of net benefit, decision-curve analysis, and prospective validation.

The primary neurocognitive outcome must be chosen in advance. Postoperative delirium, delayed neurocognitive recovery, and later postoperative neurocognitive disorder should be analyzed separately. Delirium through day 7 is a reasonable primary binary outcome for an initial study and can be analyzed with the logistic model above; repeated delirium status requires a generalized mixed model. Day-30 cognitive change and later outcomes should use continuous or generalized mixed models appropriate to their scale and sampling schedule. The outcomes should not be pooled indiscriminately. Longer follow-up introduces attrition, intercurrent illness, and a larger contribution from underlying neurodegeneration.

Sample-size planning for the initial human study should be based on the prespecified reserve-by-load interaction rather than on main effects alone. Because delirium is binary and reserve and load are continuous, power should be estimated by simulation under the planned logistic model. The simulation should specify the expected delirium incidence, the distributions and correlation of reserve and load, measurement reliability, the smallest interaction judged meaningful, covariates, center effects, and 10%–15% loss to follow-up or unusable measurements. We propose a two-sided alpha of 0.05 and 80%–90% power. If recruitment cannot meet the resulting estimate, the study should be framed as a pilot or derivation cohort, with emphasis on effect estimation rather than confirmation. External validation and nested imaging or biomarker studies will require separate calculations.

Incremental value should be tested against a reference model that contains age, sex, education, frailty, baseline cognition, vascular disease, depression, sleep disorder, medication burden, and any perioperative predictors not already used to construct the selected Lᵢ. Surgical severity and anesthetic exposure are handled either as load components or as covariates, never both. Evaluation should include likelihood-ratio testing, calibration, discrimination, internal validation, and external validation. A change in the area under the receiver operating characteristic curve is not enough on its own. Missing data, informative loss to follow-up, and center effects also need explicit treatment.

Mediation analysis follows the interaction analysis. With repeated, time-ordered measurements, a longitudinal model can test whether reserve and load predict postoperative clearance change, whether that change precedes inflammation or neural injury, and whether the trajectories relate to cognition. Because clearance and inflammation may influence each other, a single postoperative biomarker panel cannot establish mediation.

## 9 Preclinical strategy and mechanistic discrimination

Animal studies allow tighter control of exposure and tissue-level measurement. A factorial design could combine young and aged animals, sham and standardized surgery, graded mechanical-fluid load, and manipulation of a candidate pathway. Outcomes would include glymphatic influx and efflux, drainage to deep cervical lymph nodes, aquaporin-4 polarization, neuroinflammation, and behavior at prespecified times. Baseline drainage should be measured before surgery whenever the method permits, so that reserve is observed rather than inferred from the postoperative result.

PIEZO1 experiments also need cell specificity. Manipulation in lymphatic endothelium may not reproduce the effects seen in microglia, neurons, or vascular endothelium. Direction matters as well. Too little and too much mechanosensitive signaling may both be harmful, depending on the amplitude and duration of force, age, and cellular setting. The design should not assume a simple linear dose-response relationship.

A particularly informative experiment would apply the same controlled load to animals with measured differences in baseline drainage and then determine whether changing reserve before surgery shifts the load-response curve. Improvement in both drainage and cognition after a clearance-directed intervention would support the model. If PIEZO1 manipulation has no effect but the reserve-load interaction remains, other force sensors should be examined.

## 10 Testable predictions

Prediction 1—Human baseline. Clearance-reserve measures obtained 7–30 days before elective major noncardiac surgery will account for variation in delirium through day 7 and delayed neurocognitive recovery at day 30 after adjustment for established baseline risk factors and perioperative predictors not used to construct the selected load measure.

Prediction 2—Interaction. The association of mechanical-fluid or inflammatory load with cognitive outcomes will be stronger toward the lower end of the continuously modeled reserve distribution. This should be demonstrated within a defined surgical stratum before extending the analysis to cardiac or intracranial surgery.

Prediction 3—Recovery trajectory. Lower baseline reserve combined with greater load will be associated with a larger early postoperative change and slower recovery in clearance-related measures across the first 72 h and through day 30. Recovery slope should carry more information than a single postoperative value.

Prediction 4—Mechanotransduction in aging. When load is standardized, aged animals with poorer measured baseline drainage will show stronger or more persistent mechanosensitive signaling and clearance dysfunction than young animals. Measurements should cover the early hours and subsequent postoperative days.

Prediction 5—PIEZO1. Cell-specific PIEZO1 manipulation will change meningeal lymphatic drainage or brain-cerebrospinal fluid exchange after surgery. Cognitive improvement is expected only when the timing and direction of manipulation improve clearance without harmful vascular or immune effects.

Prediction 6—Reserve enhancement. Improving clearance before surgery or during early recovery will move the load-response curve toward greater tolerance in aged animals. A human prophylactic trial should follow validation of a measurable reserve phenotype and begin with a clearly defined treatment period, such as the first 72 postoperative hours.

## 11 Biopsychosocial and clinical context

Clearance biology operates within a broader clinical setting. Education and cognitive reserve influence how neural injury becomes clinically visible. Frailty, depression, anxiety, disturbed sleep, chronic pain, sensory loss, social support, mobility, nutrition, and medication burden can affect baseline testing, inflammatory state, perioperative exposure, and detection of cognitive change (2, 3, 34). Their statistical role should follow a prespecified causal diagram. Depending on the question, they may be confounders, effect modifiers, or parallel forms of reserve. Folding all of them into clearance reserve would blur rather than strengthen the construct.

Sleep illustrates this overlap. Experimental work shows that sleep can promote metabolite clearance (61), but poor sleep before surgery may also alter cognitive performance, while postoperative sleep disruption may add inflammatory and cognitive stress. Sleep is therefore better treated as a determinant and modifier than as a direct clearance marker. Pain and sedative exposure require similar care because each can affect cognition through pathways that do not depend on clearance.

Some postoperative cognitive syndromes will be dominated by other causes. Stroke, severe hypoxia, metabolic disturbance, direct neural injury, withdrawal, infection, and medication toxicity can outweigh the proposed pathway in an individual patient. These conditions should be identified as competing explanations rather than fitted into the reserve-load sequence.

PND is an umbrella outcome rather than a single etiologic entity, so clearance reserve is unlikely to contribute equally in every case. In inflammation-dominant states, such as major tissue injury or perioperative infection, baseline reserve may influence how long cytokines, cellular debris, and metabolic products persist. In cardiac surgery, reserve may modify secondary injury associated with bypass-related inflammation, venous congestion, or impaired pulsatility, but it cannot account for a focal embolic stroke. During intracranial surgery, direct tissue injury and disruption of cerebrospinal fluid pathways may become the dominant load and may also alter the clearance system itself. Medication effects, withdrawal, electrolyte disorders, hypoglycemia, or severe hypoxia can produce cognitive change through pathways that involve clearance only in part. Studies should therefore define the suspected etiologic stratum before analysis, use load variables suited to that stratum, and test whether the reserve-load interaction changes across strata rather than assuming one effect size for all PND.

## 12 Repeated surgery, aging, and long-term cognitive decline

Current evidence does not show consistently that repeated surgery or anesthesia accelerates brain aging or causes dementia. Observational studies have reached different conclusions, and estimates change when surgical indication and baseline health are considered. A large propensity-matched cohort found no increase in five-year incident dementia after common elective noncardiac surgery (33). Long-term associations are easily confounded by the illness that led to surgery, pain, mobility, vascular disease, selection for treatment, and preclinical neurodegeneration.

Lifetime surgical exposure is therefore a separate research question. It would require time-varying analysis of cumulative load, intervening illness, cognitive trajectory before each operation, and the completeness of recovery afterward. The reserve model raises the possibility that incomplete recovery after one operation may reduce capacity before the next. At present, that remains a prediction to test.

## 13 Translational implications

Current measurements cannot yet determine treatment for an individual patient. DTI-ALPS, perivascular-space burden, cerebrospinal fluid pulsatility, and circulating neural-injury markers describe different aspects of biology and cannot be substituted for one another. Before a research measure becomes a clinical label, its validity, reproducibility, calibration, and net benefit must be shown.

Within the theory, treatment is organized around preserving reserve, reducing perioperative load, and supporting recovery. Preoperative care would address reversible contributors to poor reserve or poor cognitive recovery, including sleep disturbance, dehydration, vascular instability, infection, and unnecessary psychoactive or anticholinergic medication. Intraoperative management would limit avoidable load by maintaining oxygenation and carbon dioxide near the patient's physiological range, preventing prolonged hypotension and marked venous congestion, avoiding unnecessary airway pressures, and using fluid and anesthetic strategies that do not add further physiological stress. Early postoperative care would combine regular delirium assessment with orientation, sleep protection, adequate opioid-sparing analgesia, early mobilization, infection control, and correction of hypoxemia or metabolic disturbance. These measures are consistent with perioperative brain-health practice, but the model has not shown that their benefit is mediated by improved clearance (2, 3).

If the model is validated, clinical studies could test whether patients with less measured reserve or greater recorded load gain more from intensified delirium prevention, hemodynamic management, sleep protection, or postoperative observation. Treatment-effect analyses would need to be prespecified within defined surgical and etiologic strata. Direct clearance-oriented approaches, including enhancement of meningeal lymphatic drainage, restoration of aquaporin-4 organization, or manipulation of PIEZO1 signaling, remain preclinical. They should not be offered as perioperative treatment until target engagement, safety, dose, timing, and cognitive benefit have been demonstrated. The immediate therapeutic value of the theory is therefore to organize research around who may benefit from established brain-health measures, while keeping experimental clearance interventions separate from current care.

## 14 Boundary conditions, limitations, and falsification

Important parts of the proposed chain remain untested in humans. No study has yet linked preoperative clearance measures, recorded perioperative mechanical load, serial postoperative clearance, and cognition in one cohort. Available imaging measures are indirect, while much of the molecular-cellular domain comes from animal work. The contribution of clearance may also be small when stroke, severe hypoxia, direct neural injury, infection, or medication toxicity dominates. Cardiac and intracranial surgery need separate models.

The theory also overlaps with earlier accounts of glymphatic-meningeal lymphatic imbalance in PND, including the idea that inflammatory burden can exceed central clearance capacity (23). Its scientific value will come from measurement and quantitative testing rather than from new terminology.

Mechanotransduction is retained as a secondary research program despite the limited direct perioperative evidence. The rationale is that the proposed load construct includes a distinct mechanical-fluid domain, while meningeal lymphatic and vascular compartments are force responsive and provide testable candidate sensors (45–52). This pathway is not required to define clearance reserve or to support the main reserve-by-load interaction. If mechanical exposure cannot be linked reproducibly to clearance-related responses, the mechanotransduction branch can be discarded without invalidating the broader model.

The criteria for rejection follow the level of the hypothesis. The core model would be weakened substantially if a preoperative multimodal phenotype cannot be reproduced, adds no information beyond established predictors, or shows no consistent interaction with load in adequately powered prospective studies. A lack of association between postoperative clearance trajectories and neuroimmune or cognitive outcomes would further argue against it. The mechanotransduction branch would be rejected if measured mechanical exposure does not alter clearance-related signaling or physiology. The PIEZO1 hypothesis would fail if cell-specific manipulation does not change the relevant drainage, inflammatory, or cognitive findings under controlled perioperative conditions. A negative result at one mechanistic level does not by itself settle the higher-level interaction question.

A useful model should reduce uncertainty. Reserve must therefore remain continuous, measurable before the outcome, and open to external validation. Clinical cutoffs and prophylactic treatment rules belong only after those requirements have been met.

## 15 Conclusion

Differences in perioperative cognitive recovery may reflect a mismatch between the clearance capacity present before surgery and the load imposed during and after the operation. This proposal can be examined with measurements already available in research settings: assess reserve before surgery, record load over time, follow postoperative clearance recovery, and define cognitive outcomes within accepted windows. Mechanotransduction and PIEZO1 provide testable biological routes, but the main question is broader. Clearance reserve will be useful only if it adds information beyond established clinical, inflammatory, vascular, and cognitive predictors and if its interaction with load can be reproduced in new cohorts.

## Data Availability

The original contributions presented in the study are included in the article/Supplementary Material, further inquiries can be directed to the corresponding author.
